# miR-352 participates in the regulation of trypsinogen activation in pancreatic acinar cells by influencing the function of autophagic lysosomes

**DOI:** 10.18632/oncotarget.24220

**Published:** 2018-01-13

**Authors:** Zonggong Song, Yongming Huang, Chao Liu, Ming Lu, Zhituo Li, Bei Sun, Weihui Zhang, Dongbo Xue

**Affiliations:** ^1^ Department of General Surgery, The First Affiliated Hospital of Harbin Medical University, Harbin, China; ^2^ Department of Surgery, David Geffen School of Medicine, University of California at Los Angeles, Los Angeles, CA, USA

**Keywords:** acute pancreatitis, miR-352, LAMP2, CTSL1, autophagic lysosomes

## Abstract

This study was performed to screen miRNAs and mRNAs that are differentially expressed during trypsinogen activation in acute pancreatitis and to verify their role in the process of trypsinogen activation. The function enrichment analysis showed that the functions of miR-352 and its regulatory targets lysosome-associated membrane protein 2 (LAMP2) and cathepsin L1 (CTSL1) were lysosome related. The results of the verification experiment showed that in the TLC-S-treated AR42J (pancreatic cell line) cells, miR-352 expression increased, expression levels of LAMP2 and CTSL1 were significantly reduced, trypsinogen activation was increased, and the autophagy pathway was blocked. In the miR-352 mimic-transfected cells, miR-352 expression increased, expression levels of LAMP2 and CTSL1 were significantly reduced, trypsinogen activation was increased, intracellular lysosomal pH increased, cathepsins L activity decreased and the amount of autophagolysosomes increased. In the miR-352 inhibitor-transfected cells, miR-352 expression was reduced, expression levels of LAMP2 and CTSL1 were significantly increased, trypsinogen activation was decreased, intracellular lysosomal pH decreased, cathepsins L activity increased and the amount of autophagolysosomes decreased. In the process of taurolithocholic acid 3-sulfate (TLC-S) induced trypsinogen activation, overexpression of miR-352 could down-regulate LAMP2 and CTSL1, resulting in the dysfunction of autophagic lysosome. Thus, the autophagy pathway was blocked, and trypsinogen activation was enhanced.

## INTRODUCTION

Acute pancreatitis (AP) has high morbidity and mortality rates. Although its pathogenesis is not yet fully elucidated, it is widely accepted that the self-digestion of cells caused by the trypsinogen activation in pancreatic acinar cells is the initiating factor for all types of AP and has also become an important indicator in determining the occurrence of AP [1, 2]. In recent studies, abnormality in the autophagy pathway of pancreatic acinar cells led to the activation of pancreatic acinar cell vacuoles and the activation of trypsinogen [3, 4]. The dysfunction of autophagosomes, lysosomes, and mitochondria is an important factor in the pathogenesis of AP [4] .

Autophagy (mainly macroautophagy) is the lysosome-mediated process for intracellular degradation and for the recycling of organelles, longevity proteins, and lipids. The autophagosome is formed first and engulfs the substances to be degraded [5]. The autophagosome is ultimately fused with a lysosome to form an autophagic lysosome, in which lysosomal hydrolases, such as cathepsin B (CTSB) and cathepsin L (CTSL, also known as CTSL1), will degrade the substances [3, 6, 7]. Ohmuraya et al. [8] performed an experiment showing that Spink3 knockout mice exhibited excessive autophagy, which subsequently induced the abnormal activation of trypsinogen. Yang et al. [9] believed that in the rats with severe AP induced by the injection of sodium taurocholate into the pancreatic duct, the NF-κB pathway was activated, which promoted autophagosome formation and trypsinogen activation. Autophagosome formation is mediated by a series of highly evolutionally conserved autophagy-related proteins, and the autophagy process is highly dependent on the function of lysosomes [7] . Mareninova et al. [3] showed that during the onset of AP, the autophagic pathway was blocked and the lysosomal hydrolases CTSB and CTSL1 were less mature with lower activity, resulting in increased activation of trypsinogen in the autophagic lysosome. Orlichenko et al. [10] indicated that in bombesin-induced AP, ARF1 could promote the activation of trypsinogen by promoting the transportation and processing of the precursor of lysosomal cysteine protease CTSB and the maturation of the autophagosome.

The genetic regulation mechanisms of the autophagic pathway and trypsinogen activation have been widely reported [11–13]. In the post-genomic era, the regulating role of miRNA in a number of biological processes has increasingly caused concern. Studies have reported that miRNA plays an important role in AP. For example, Hu et al. [14] suggested that the overexpression of miR-19b could induce the necrosis of pancreatic acinar cells by inhibiting the expression of its target genes. Ma et al. [15] found that miR-21 up-regulation could promote the necrosis of pancreatic acinar cells by activating the protein kinases RIP1 and RIP3. Fu et al. [16] showed that during the onset of AP, miR-29 was increased significantly, and the apoptosis of pancreatic acinar cells was promoted by increasing the expression of the TNFRSF1A gene.

Therefore, this study aimed to investigate the regulatory role of miRNA on trypsinogen activation, to identify the miRNA-mRNA pairs that are coexpressed during trypsinogen activation in pancreatic acinar cells using miRNA microarray detection, mRNA microarray detection, and bioinformatics integration analysis. The biological functions of these miRNAs were verified using loss-of-function and gain-of-function experiments, and the resulting information is expected to provide a new target for a mechanism study and for the treatment of AP.

## RESULTS

### Detection of miRNA and mRNA with differential expression and integration analysis

**Figure 1:**
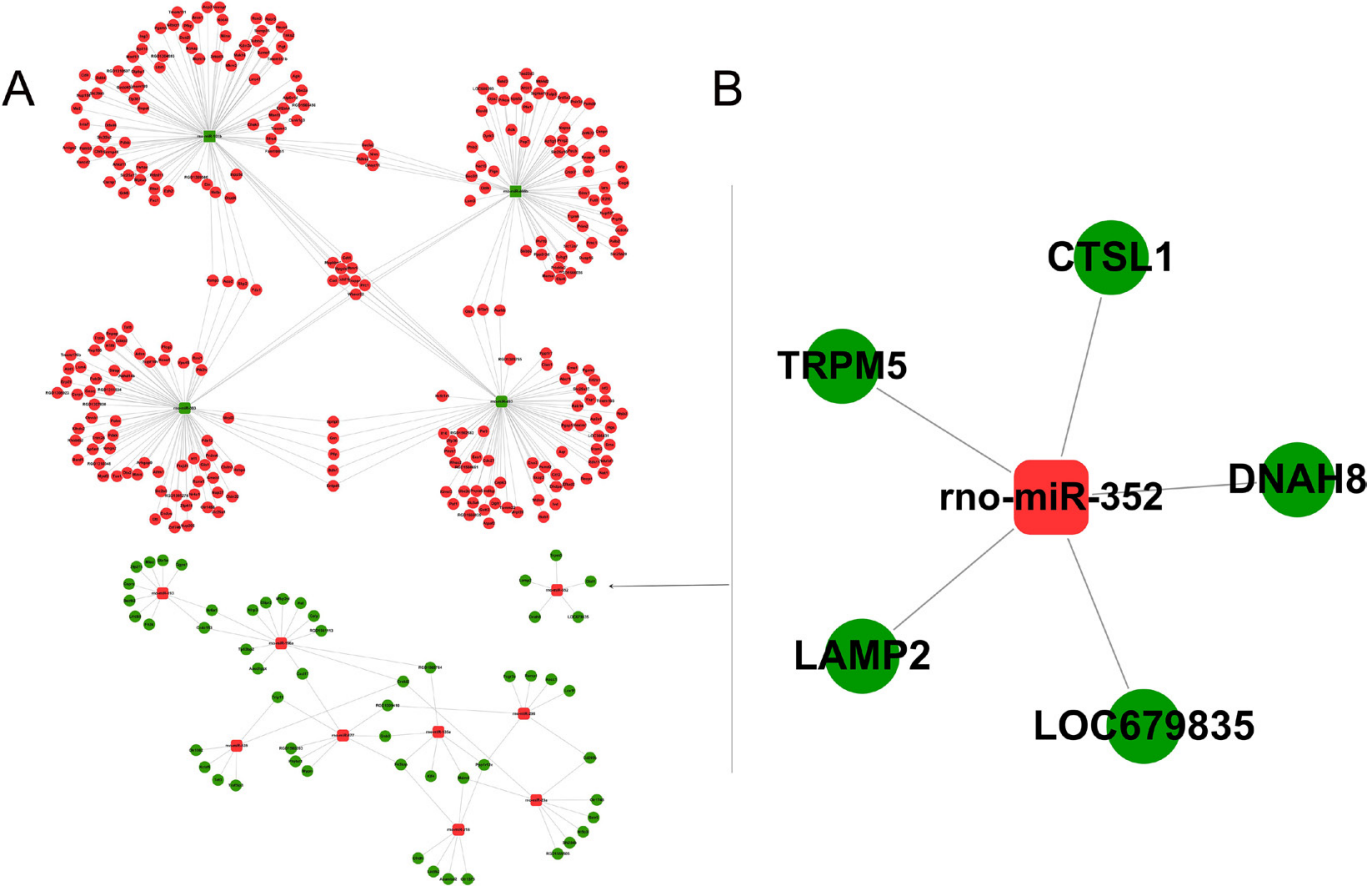
Integrated analysis of miRNA-mRNA co-expression The miRNA-mRNA regulatory network showing the differential expression in the rat pancreatic AR42J cells treated with TLC-S. The square nodes represent miRNA, and the circular nodes represent mRNA. Red indicates up-regulation, and green indicates down-regulation. (**A**) Overall regulatory network, (**B**) miR-352 regulatory network.

**Figure 2:**
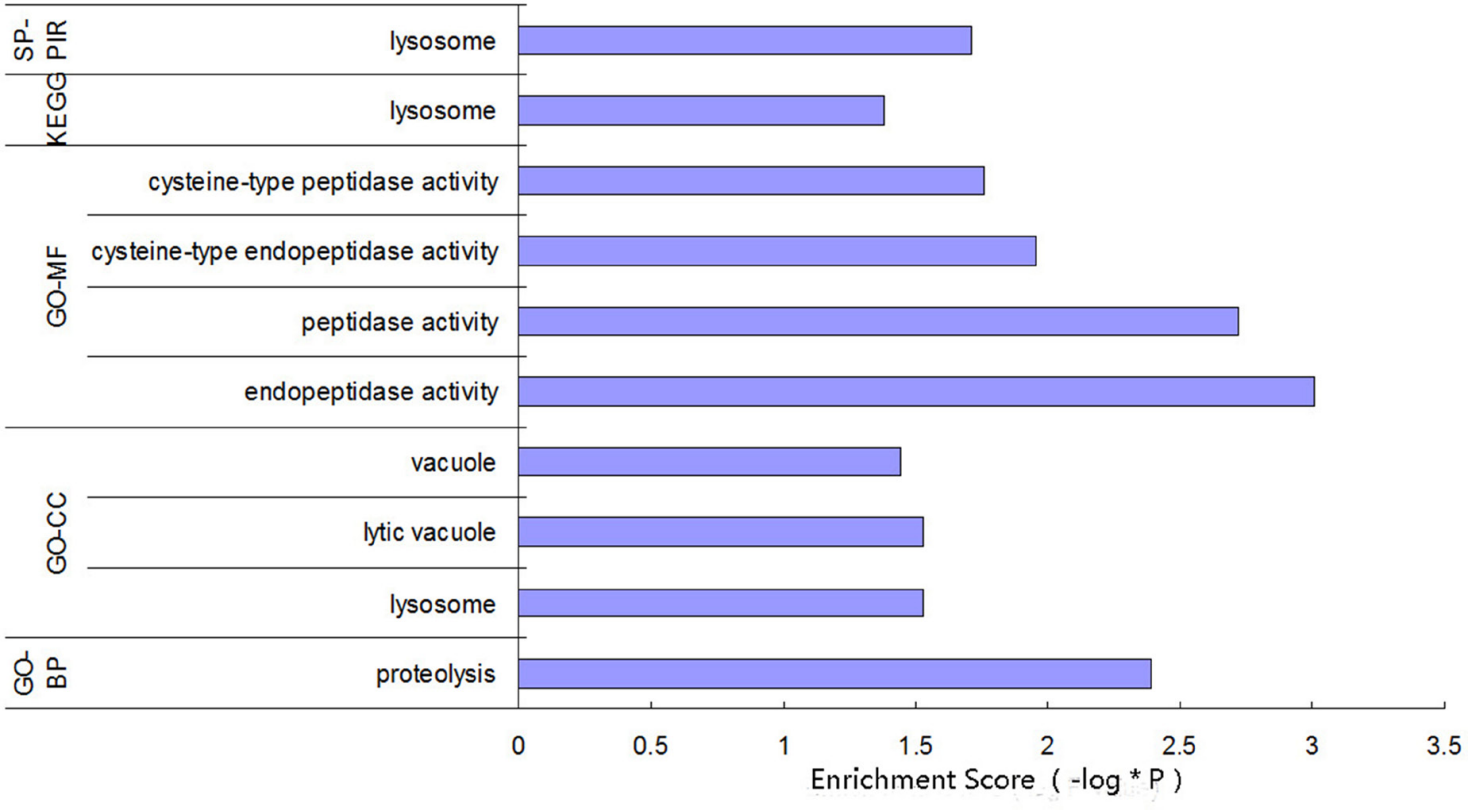
Function enrichment analysis for the miR-352-regulated genes

Based on the preset threshold, the miRNA and mRNA with differential expression were identified, and the pairs with a regulation relationship, as indicated by the bioinformatic prediction, were saved, resulting in 337 relationship pairs. The constructed regulatory network (Figure 1A) contains 13 differentially expressed miRNAs and 337 differentially expressed mRNAs, in which 4 miRNAs were down-regulated and 283 of the related corresponding mRNAs were up-regulated; 9 miRNAs were up-regulated, and 54 of the related corresponding mRNAs were down-regulated. The function enrichment analysis of the mRNA regulated by each miRNA showed that the functions of miR-352 in the Swiss-Prot and ProteinInformation Resource KEYWORDS (SP-PIR-KEYWORDS) analysis were mainly enriched in “lysosome”. The functions found using Kyoto Encyclopedia of Genes and Genomes( KEGG) analysis were mainly enriched in “Lysosome (rno04142)”. The functions found using gene ontology molecular function (GO-MF) analysis were mainly enriched in “proteolysis (0006508)”, “peptidase activity (0008233)”, “cysteine-type endopeptidase activity (0004197)”, and “cysteine-type peptidase activity (0008234)”. The gene ontology cellular components (GO-CC) analysis were mainly enriched in “lysosome (0005764)”, “lytic vacuole (0000323)”, and “vacuole (0005773)”. The functions found using gene ontology biological process (GO-BP) analysis of biological processes were mainly enriched in “proteolysis (0006508)”, as shown in Figure 2. Therefore, miR-352 should be the target miRNA of interest for our research.

**Figure 3:**
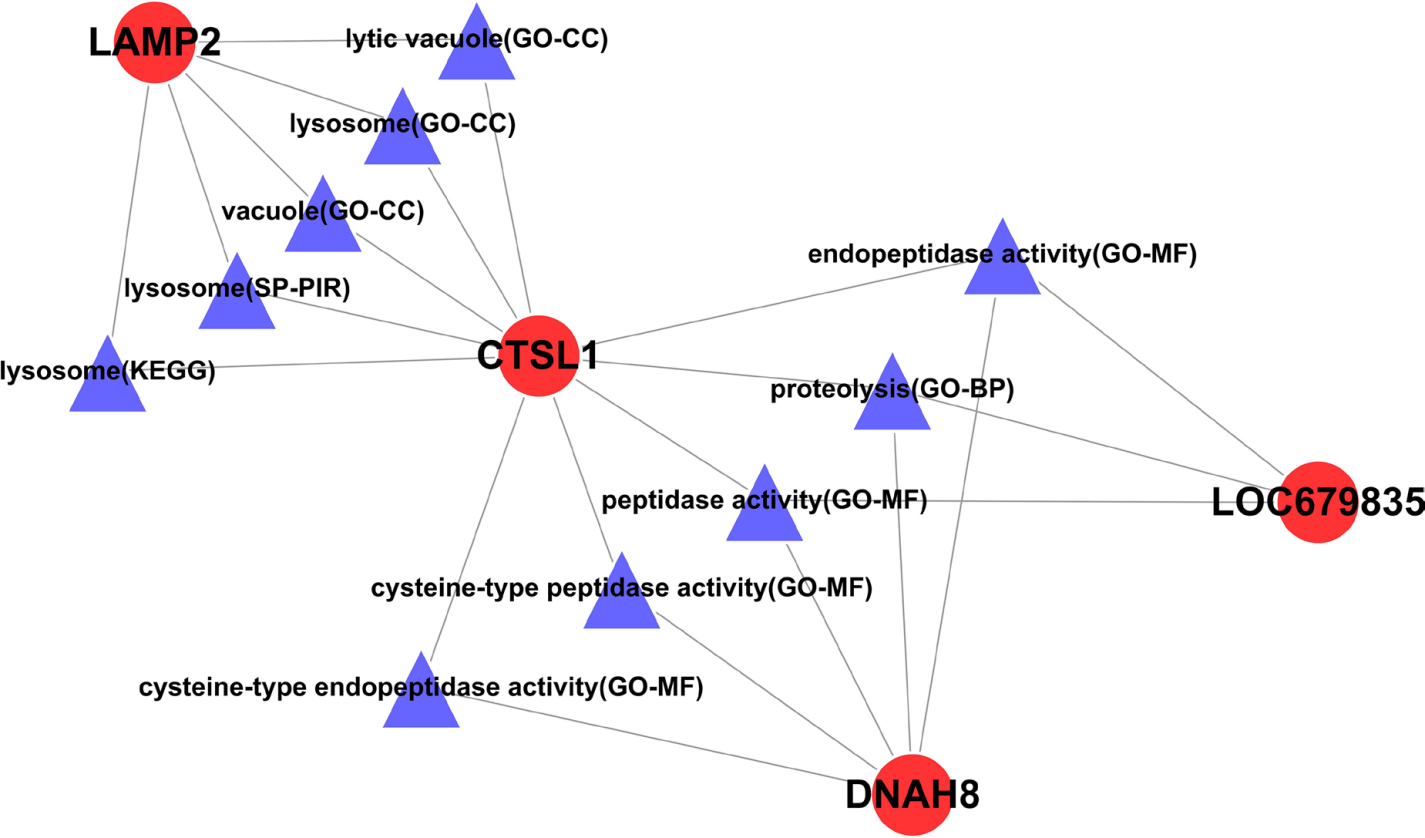
The rno-miR-352-regulated genes and their functional relationship network Circular nodes represent mRNA, and triangular nodes represent the enriched function.

As shown in Figure 1B, miR-352 regulates the expression of five genes, including LAMP2, DNAH8, CTSL1, TRPM5, and LOC679835. Further function analysis (Figure 3) showed that the functions of LAMP2 and CTSL1 were enriched in “lytic vacuole (GO-CC)”, “lysosome (GO-CC)”, “vacuole (GO-CC)”, “lysosome (SP-PIR)”, and “lysosome (SP-PIR)”. Therefore, LAMP2 and CTSL1 should be the target mRNA of interest for our research.

### Real-time polymerase chain reaction (PCR) results for rno-miR-352 in each group after the miR-352 intervention

**Figure 4:**
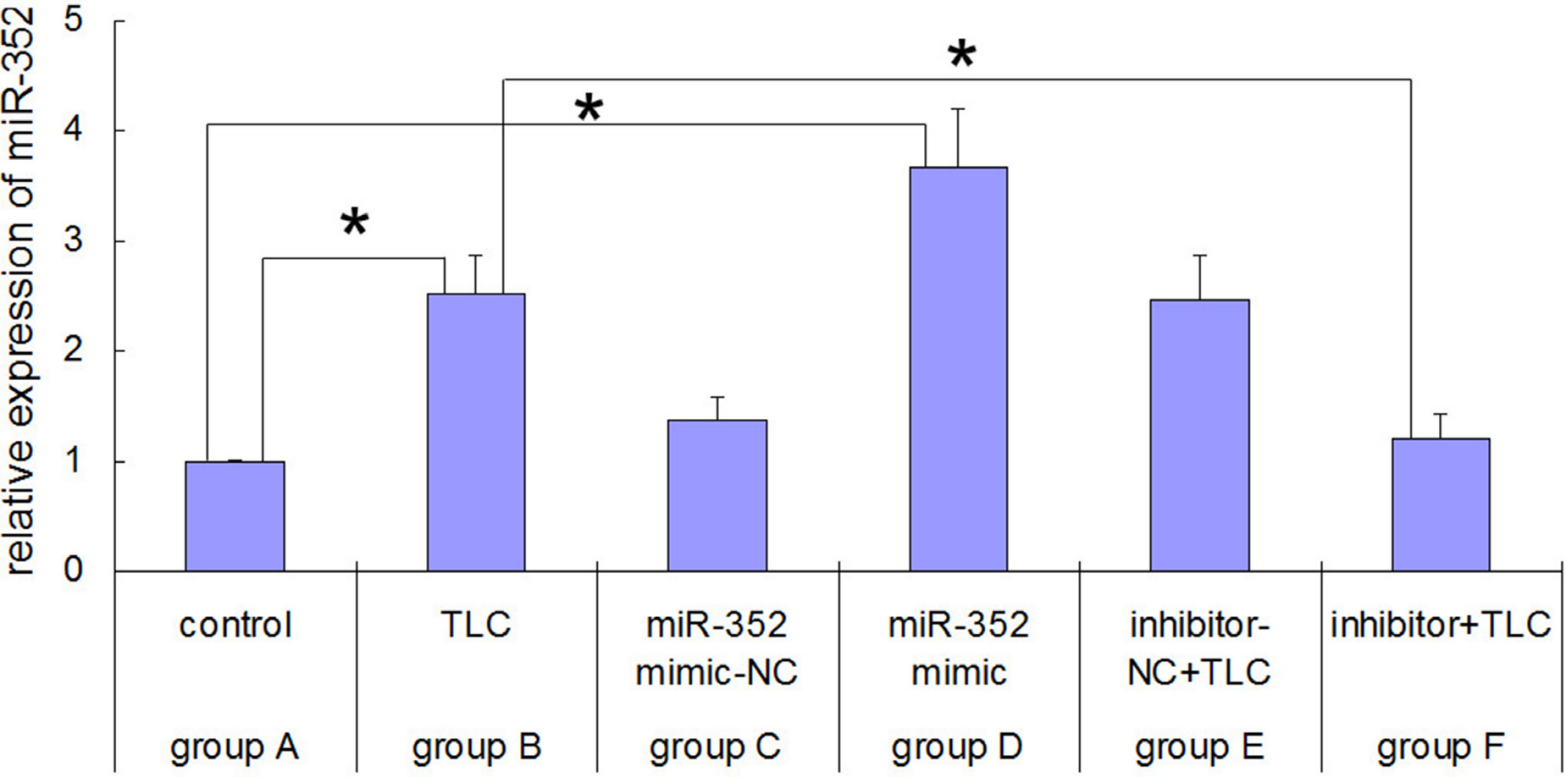
Real-time PCR results for rno-miR-352 in each group before and after rno-miR-352 intervention ^*^P < 0.05 versus control.

As shown in Figure 4, after the AR42J cells were treated with taurolithocholic acid 3-sulfate (TLC-S), the expression of rno-miR-352 was significantly higher than that of the normal AR42J cells (2.526 ± 0.363 vs. 1.0 ± 0.017, *P* < 0.05), which is consistent with the microarray results. After the normal AR42J cells were transfected with an rno-miR-352 mimic, the expression of rno-miR-352 was significantly higher than that of the normal AR42J cells (3.663 ± 0.538 vs. 1.0 ± 0.017, *P* < 0.05). After the TLC-S treated AR42J cells were transfected with an rno-miR-352 inhibitor, the expression of rno-miR-352 was significantly lower than that of the TLC-S-treated AR42J cells (1.217 ± 0.226 vs. 2.526 ± 0.363, *P* < 0.05). The transfection of the rno-miR-352 negative control showed no significant impact on the expression of rno-miR-352 in the corresponding group.

### Results of trypsinogen activation in each group after the miR-352 intervention

**Figure 5:**
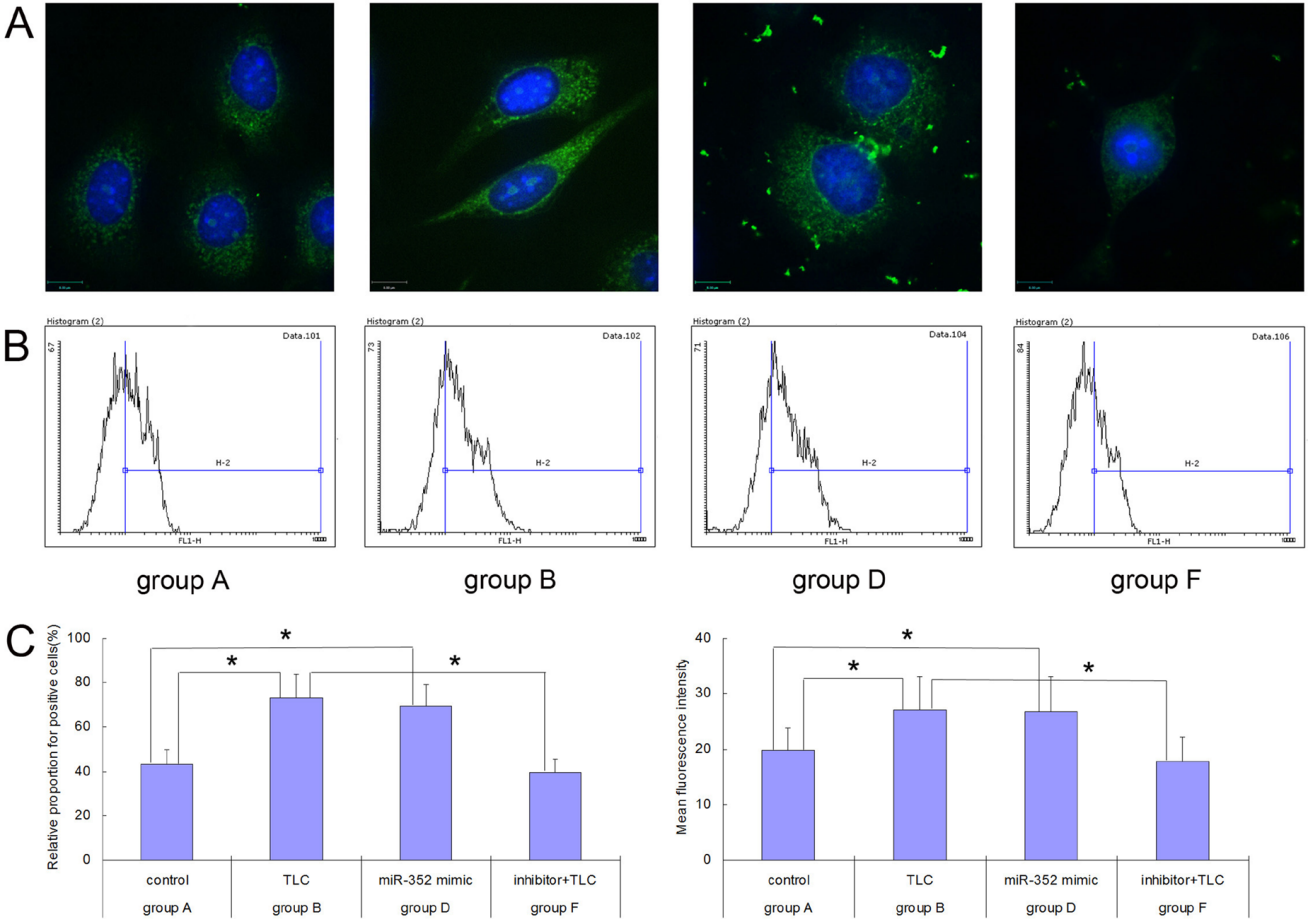
Trypsinogen activation detected in each group of AR42J cells before and after the rno-miR-352 intervention (**A**) Image from laser confocal microscopy. (**B**) Flow cytometry results. (**C**) Chart of statistics. ^*^P < 0.05.

BZiPAR is a specific substrate for serine protease. When BZiPAR is cleaved into two oligopeptides and reacted with activated trypsin, green fluorescence will be emitted. In this experiment, a quantitative study was performed using flow cytometry. The activation level of trypsinogen was evaluated by the relative proportion of the green fluorescence-positive cells and the mean fluorescence intensity (Figure 5C). The results showed that the activation of trypsinogen in the TLC-S-treated cells was significantly higher than that of the control group (relative proportion for positive cells: 73.25% ± 10.46% vs. 43.12% ± 6.86%, *P* < 0.05; mean fluorescence intensity: 27.05±6.07 vs. 19.76 ± 3.97, *P* < 0.05). Although not treated with TLC-S, the AR42J cells transfected with rno-miR-352 mimic showed significantly increased trypsinogen activation compared with that of the control group (relative proportion for positive cells: 69.47% ± 9.72% vs. 43.12% ± 6.86%, *P* < 0.05; mean fluorescence intensity: 26.69 ± 6.38 vs. 19.76 ± 3.97, *P* < 0.05). For the TLC-S treated group, the trypsinogen activation after transfection with the rno-miR-352 inhibitor was significantly lower than that of the non-transfected group (relative proportion for positive cells: 39.49% ± 6.07% vs. 73.25% ± 10.46%, *P* < 0.05; mean fluorescence intensity: 17.83 ± 4.42 vs. 27.05 ± 6.07, *P* < 0.05). The image from laser confocal microscopy also showed this change trend (Figure 5A).

### Western blot results for LAMP2 and CTSL1 proteins in each group after the miR-352 intervention

**Figure 6:**
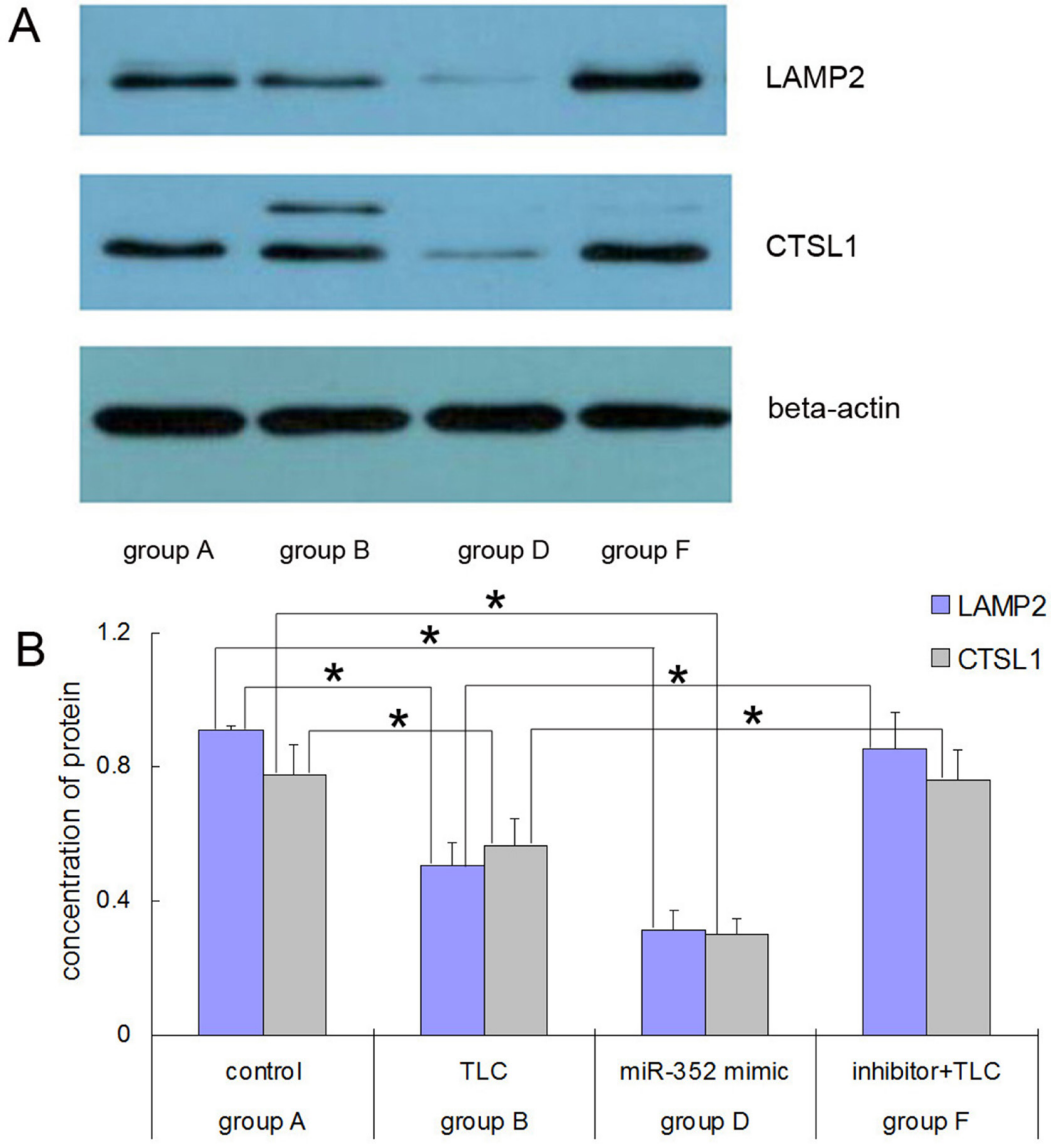
Western blot results for the LAMP2 and CTSL1 proteins in each group after rno-miR-352 intervention (**A**) Electrophoresis. (**B**) Chart of statistics. ^*^P < 0.05.

As shown in Figure 6, the expression levels of LAMP2 and CTSL1 proteins in the TLC-S-treated cells were significantly lower than those in the control group (LAMP2: 0.5059 ± 0.0687 vs. 0.9123 ± 0.0128, *P* < 0.05; CTSL1: 0.5675 ± 0.0792 vs. 0.7753 ± 0.0921, *P* < 0.05). The expression levels of LAMP2 and CTSL1 proteins in the AR42J cells transfected with the rno-miR-352 mimic were also significantly lower than those in the control group (LAMP2: 0.3123 ± 0.0579 vs. 0.9123 ± 0.0128, *P* < 0.05; CTSL1: 0.3008 ± 0.0482 vs. 0.7753 ± 0.0921, *P* < 0.05). The expression levels of LAMP2 and CTSL1 proteins in the AR42J cells transfected with the rno-miR-352 inhibitor were significantly higher than those in the TLC-S-treated cells without transfection (LAMP: 0.8556 ± 0.1062 vs. 0.5059±0.0687, *P* < 0.05; CTSL1: 0.7594 ± 0.0902 vs. 0.5675 ± 0.0792, *P* < 0.05).

### Results for the blockage role of miR-352 in autophagy pathway in AR42J cells

**Figure 7:**
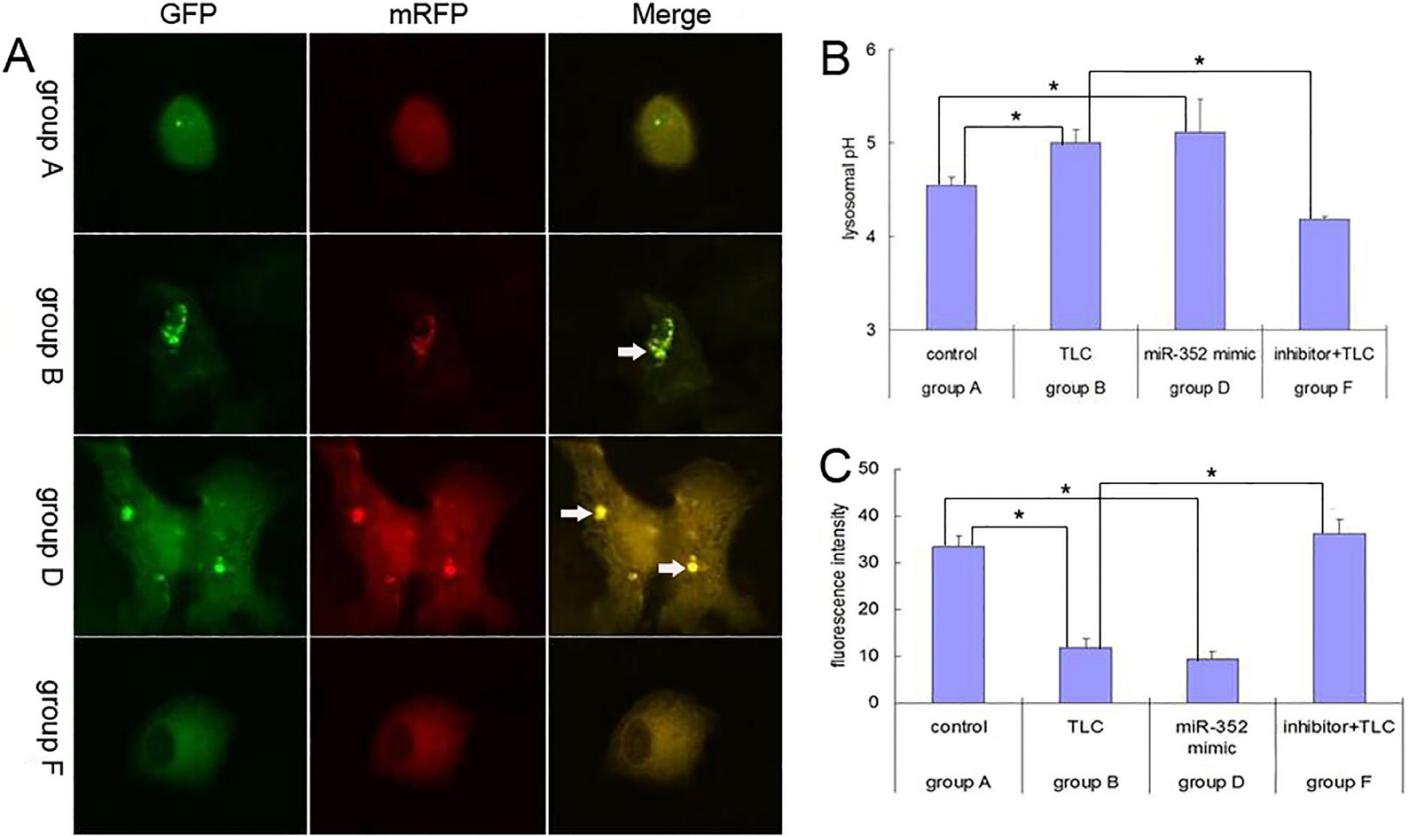
Results for the blockage role of miR-352 in autophagy pathway in AR42J cells (**A**) Autophagic flux in AR42J cells. Arrows indicate autophagolysosomes. (**B**) Intracellular lysosomal pH. (**C**) Cathepsins L activity in AR42J cells is represented by the fluorescence intensity. ^*^P < 0.05.

Intracellular lysosomal pH can affect cathepsins L activity. The results showed that the lysosomal pH in the TLC-S-treated cells was significantly higher than that of the control group and the group transfected with the rno-miR-352 inhibitor (5.001 ± 0.144 vs. 4.539 ± 0.097, *P* < 0.05; 5.001 ± 0.144 vs. 4.177 ± 0.03, *P* < 0.05), the lysosomal pH in the AR42J cells transfected with the rno-miR-352 mimic was also significantly higher than that of the control group (5.115 ± 0.35 vs. 4.539 ± 0.097, *P* < 0.05) (Figure 7B). The results of cathepsins L activity assay showed that, compared with the control group and the group transfected with the rno-miR-352 inhibitor, cathepsins L activity in the AR42J cells treated with TLC-S was significantly decreased (mean fluorescence intensity: 11.847 ± 1.994 vs. 33.447 ± 2.149, *P* < 0.05; 11.847 ± 1.994 vs. 36.251 ± 3.051, *P* < 0.05), the cathepsins L activity in the AR42J cells transfected with the rno-miR-352 mimic was also significantly decreased compared with the control group after treated with TLC-S for 40 min (9.37 ±1.776 vs.33.447 ± 2.149) (Figure 7C). The results of analysis of autophagic flux showed that the amount of autophagolysosomes increased significantly in the AR42J cells after treated with TLC-S or transfected with the rno-miR-352 mimic (Figure 7A). These results confirmed that LAMP2 and CTSL1 are the target of miR-352 and indicated that increase of miR-352 expression leads to the blockage of autophagy pathway *in vitro*.

## DISCUSSION

For a long time, both experimental research and clinical pathological studies showed that AP has an obvious pathological feature in which many large vacuoles formed and then accumulated in pancreatic acinar cells [3, 6, 17]. Recent studies have found that the vacuoles in AP acinar cells originate from autophagy, and the intracellular activation of trypsinogen in early AP occurs inside these large vacuoles [3, 6] . These findings suggest that autophagy may play an important role in the process of intracellular trypsinogen activation. Some studies have suggested that miRNA can regulate the autophagic pathway by regulating the related genes to play a role in the AP process. Zhu et al [18] found that in AP, miR-141 could bind to the miRNA 3’UTR of high mobility group box 1 (HMGB1) protein and then inhibit the expression of HMGB1 at the protein level, thus also reducing Beclin-1. This finding suggested that miR-141 could inhibit autophagosome formation through the HMGB1/Beclin-1 pathway. Gao et al. [19] showed that the expression of rno-miR-148b-3P was down-regulated in AR-42J cells and that the expression of its inhibiting genes was up-regulated, thus promoting the autophagy process through the insulin signaling pathway.

In our study using the trypsinogen activation model induced by using TLC-S, 13 differentially expressed miRNAs and 337 differentially expressed mRNAs were found using biological microarray detection. The correspondence between the miRNA and the mRNA of the target genes was predicted using the Microcosm software to construct the miRNA-mRNA regulatory network. Bioinformatics analysis found that the functions of the five genes of LAMP2, CTSL1, DNAH8, LOC679835, and TRPM5 regulated by miR-352 were mainly enriched in “lysosome” (SP-PIR, KEGG), and GO-CC analysis indicated that these genes were mainly located at “vacuole”, “lytic vacuole”, and “lysosome”, suggesting that miR-352 may be involved in the regulation of lysosomal functions. Recently, Tao et al. [20] also confirmed this finding. In the case of occlusion in the middle cerebral artery, miR-352 in the primary cortical neurons showed negative feedback regulation of the lysosomal enzyme hexosaminidase B (HEXB), thereby promoting cell death.

The results of our study indicated that the expression of miR-352 was increased in TLC-S-stimulated rat pancreatic acinar AR42J cells, whereas the expression levels of LAMP2 and CTSL1 were suppressed and trypsinogen activation was enhanced. In the gain-of-function experiment, after the AR42J cells without TLC-S stimulation were transfected with the miR-352 mimic, the expression of miR-352 was up-regulated compared with that in the non-transfected cells, and the expression levels of LAMP2 and CTSL1 were down-regulated, with enhanced trypsinogen activation. Thus, the transfection with the miR-352 mimic alone achieved an effect similar to that for the trypsinogen activation stimulated by TLC-S. In the loss-of-function experiment, after the TLC-S-stimulated AR42J cells were transfected with the miR-352 inhibitor, the expression of miR-352 was down-regulated compared with that in the non-transfected cells, and LAMP2 and CTSL1 were up-regulated, with reduced trypsinogen activation. Thus, the inhibition of miR-352 could reduce the TLC-S-stimulated trypsinogen activation.

LAMP2 is the main component of the lysosomal membrane in normal human pancreatic tissues [21] , and it mainly regulates the final stage of the autophagy process: the fusion of the autophagosome with lysosomes [22–24] . The consumption of LAMP2 plays a key role in the pathogenesis of early AP, which may cause the impairment of autophagy [25] . Fortunato et al. [26] indicated that in the pancreatic acinar cells of AP mice induced by alcohol combined with endotoxin, the expression of LAMP2 was lost. This change led to a disorder in the fusion of lysosomes and autophagosome and a blockage in the autophagy pathway, so a large number of the vacuoles in the cells were autophagosomes instead of lysosomes. However, some lines of evidence suggested that those vacuoles are probably autophagic lysosomes and that their accumulation was caused by the impairment of the autophagy pathway [3, 27, 28] . In either case, the consensus is that the autophagy pathway of AP is blocked, with lysosomal dysfunction. The lack of LAMP2 may cause Danon disease in patients, showing various types of cytoplasmic vacuole accumulation,including the accumulation of pancreatic acinar cells [22, 29]. Cytoplasmic vacuoles are closely related to the activation of trypsinogen particles and autophagosome formation. Additionally, the formation of cytoplasmic vacuoles is an early sign of cell and tissue damage in AP, but its exact mechanism remains unclear [17]. Thus, the overexpression of miR-352 inhibits the expression of LAMP2, causing disorders in the autophagosome-lysosome fusion, thus resulting in dysfunction in autophagic lysosomes, impairment in the autophagy pathway, and formation of a large number of vacuoles. Therefore, the activation of trypsin is enhanced, leading to the occurrence of AP.

CTSL1 is a cysteine protease and plays an important role in degradation in the lysosome, which can degrade trypsin and trypsinogen [3, 30]. Studies have found that CTSL1 reacts at site G26-G27 of trypsinogen to hydrolyze trypsinogen and also reacts at the site E82-E83 in the calcium-binding ring to hydrolyze trypsinogen and trypsin [30]. Studies have shown that the autophagy pathway in the pancreatic acinar cells of AP is blocked, with less mature CTSL1 [3]. If the process of CTSL1 maturation is blocked or if its activity is low, the degradation function of lysosomes can be decreased, resulting in autophagic dysfunction and more activated trypsin in autophagic lysosomes [3, 4]. Therefore, in the AP induced by TLC-S, the overexpression of miR-352 inhibited the expression of CTSL1, causing the dysfunction in autophagic lysosomes, with a low efficiency in clearing trypsinogen and trypsin. This change leads to the increase in trypsinogen and trypsin in the acini while increasing the activation of trypsin.

In the AP induced by TLC-S, miR-352 is up-regulated to cause the down-regulation of LAMP2 and CTSL1, resulting in autophagic lysosomal dysfunction and the blockage of the autophagy pathway. These changes cause trypsinogen activation, which is entirely consistent with our previous prediction by software, which suggested that the down-regulation of LAMP2 and CTSL1 may together promote the occurrence of AP. After the expression of miR-352 was down-regulated by the miR-352 inhibitor, LAMP2 and CTSL1 were up-regulated, and trypsinogen activation was reduced. These changes reduced the damage to the pancreatic cells in AP. Therefore, this study provides a new clue for the further study of new drugs for AP treatment using miR-352 as a target.

## MATERIALS AND METHODS

### Detection of mRNA and miRNA expression using microarrays

The rat pancreatic acinar cell line AR42J was purchased from China Collection Cell Bank, and the cells were cultured with Ham’s F12K medium containing 10% fetal bovine serum, 100 U/ml penicillin, and 100 μg/ml streptomycin in a 37°C incubator with 5% CO_2_. The cells were divided into two groups: the control group and the experimental group. The cells in the control group were not specially treated, while the cells in the experimental group were treated with 200 μM/L taurolithocholic acid 3-sulfate (TLC-S; Sigma Aldrich, St. Louis, USA) for 20 min. The concentration and reaction time of TLC-S were selected based on the reports of Gerasimenko and Voronina et al. [31, 32].

The cells were collected, and the total RNA was extracted according to the instructions for the TRIzol reagent (Invitrogen,Carlsbad, CA, USA).

Gene expression analyses were done with the Rat 12 × 135K Gene Expression Array (NimbleGen Systems, Inc., Madison, WI, USA). Preparation of cRNA from 5μg of total RNA, hybridizations, washes, and detection were done in accordance with the protocol of NimbleGen Gene Expression Analysis,the slides were scanned using the Axon GenePix 4000B microarray scanner. Expression data were normalized through quantile normalization and the Robust Multichip Average algorithm included in the NimbleScan software. All gene level files were imported into Agilent GeneSpring GX software (version 11.5.1) for further analysis. Differentially expressed genes were identified through fold change filtering.

miRNA expression analyses were done with the Affymetrix^®^ GeneChip^®^ miRNA Arrays (Affymetrix, Inc., Santa Clara,CA,USA). Poly(A) Tailing, FlashTag Ligation,Hybridization, Washing, Staining, and detection were done in accordance with the protocol of Affymetrix^®^ GeneChip^®^ miRNA Arrays scan using the Library file (http://www.affymetrix.com/support/downloads/manuals/agcc_command_console_user_guide.pdf). Expressed data were normalized using the median normalization. After normalization, differentially expressed miRNAs were identified through fold change filtering.

### Integrated analysis of miRNA-mRNA co-expression

The gene names were integrated by referring to the abbreviations in the Rat Genome Database (RGD). An mRNA with a fold change value ≥ 2.0 or ≤ 0.5 was considered differentially expressed. The differential multiples of miRNA transcription were compared. The up-regulated miRNAs and down-regulated miRNAs were identified based on a differential multiple of 1.5.

MicroCosm software was used to predict the target gene corresponding to the differentially expressed miRNA. Any case that met the criteria of either up-regulation in the miRNA microarray and down-regulation in the mRNA microarray for the predicted target gene or down-regulation in the miRNA microarray and up-regulation in the mRNA microarray for the predicted target gene was considered a potential miRNA and target gene mRNA pair.

Based on the coexpressed mRNA-miRNA pairs, the miRNA-mRNA regulatory network was constructed using Cytoscape software (Ver 2.6.3).

The function enrichment analysis for the genes regulated by each miRNA was performed using David software (http://david.abcc.ncifcrf.gov/), and then the functions of the miRNA were annotated. The specific functions of each mRNA were shown with Cytoscape software (Ver 2.6.3).

### Cell transfection experiment

The siRNA-lipoRNAiMAX mixture (Lipofectamine^®^ RNAiMAX Transfection Reagent Cat. No 13778-150, GIBCO, USA) was prepared and added to a culture well containing 800 μL of culture medium with cells. After 4–6 h of culture, the culture medium was replaced with fresh medium (containing fetal bovine serum and double antibodies), followed by continuous culturing for 48 h. The samples were divided into six experimental groups: group A, normally cultured AR42J cells; group B, non-transfected AR42J cells treated with 200 μM TLC-S for 40 min; group C, AR42J cells transfected with miR-352 mimic-NC; group D, AR42J cells transfected with an miR-352 mimic; group E, AR42J cells transfected with miR-352 inhibitor-NC and treated with 200 μM TLC-S for 40 min; and group F, AR42J cells transfected with an miR-352 inhibitor and treated with 200 μM TLC-S for 40 min. The sequence of the rno-miR-352 mimic was AGA GUA GGU UGC AUA GUA, and the sequence of rho-miR-352 inhibitor was TCC AAC CAT ACA GAC TAC (Ribobio, Guanhzhou, China).

### Real-time PCR to determine the expression of rno-miR-352

RNA was extracted from the samples using total RNA TRIzol reagent (Cat No. DP405-02, Tiangen Biotech, Beijing, China). A PrimeScript™ RT reagent Kit with gDNA Eraser (Cat No. RR047B, TaKaRa, Japan) was used for cDNA reverse transcription, with a rno-miR-352 quantitative PCR (qPCR) primer (Product ID: RmiRQP1251, GeneCopoeia, USA), rno-miR-352 qPCR primer(RmiRQP0418, GeneCopoeia,USA), U6 snRNA primer (RQP047936, GeneCopoeia, USA)。An ABI 7500 fluorescence quantitative PCR analyzer (Applied Biosystems, USA) was used, and the relative quantification of the data was performed using the 2^-△△Ct^ method.

### Flow cytometry to detect the activation of trypsinogen

After the cells were digested and collected, 1×10^6^ cells from each group were centrifuged at 1,000 rpm for 5 min. The supernatant was discarded before 500 μL of 1× phosphate-buffered saline (PBS) was added to wash the sample two times, with centrifugation at 1,000 rpm for 5 min for each wash. The cells were then resuspended with 200 μL of BZiPAR (CBZ-Ile-Pro-Arg) 2-rhodamine 110 (Molecular Probes, USA) working solution, followed by reaction at room temperature in the dark for 60 min. After the centrifugation at 1,000 rpm for 5 min, the cells were resuspended with 500 μL of 1× PBS and evaluated using flow cytometry (Calibur II, BD Bioscience, USA). The cells were collected using CellQuest software, and the experimental data were analyzed using the Flowing software program. The state of the cells was observed with FSC/SSC, and the fluorescence intensity of the cells was observed with fluorescence channel FL1.

### Laser confocal microscopy to evaluate trypsinogen activation

The cells in the confocal dish were washed with PBS 2-3 times; 150 μL of 4% paraformaldehyde was added for 15 min at room temperature to fix the cells. After being washed with PBS 2-3 times, the cells were incubated with PBS containing 2% Tween-20 for 10 min at room temperature. After being washed with PBS again 2-3 times, BZiPAR staining solution was added at room temperature in the dark for 60 min. After being washed with PBS another 2-3 times, the cells were directly observed under a laser confocal microscope (A1R, Nikon, Japan).

### Western blot to determine protein expression

The proteins were extracted from 1 × 10^7^ cells and quantified with the bicinchoninic acid (BCA) method. The protein samples were applied at 10 μg/well, and electrophoresis was performed through the stacking gel at a constant voltage of 90 V for approximately 20 min and through the separating gel at a constant voltage of 160 V. The termination of the electrophoresis was determined according to the run using pre-stained protein markers. The wet method was used for membrane transfer with the following conditions: a constant current of 300 mA, NC membrane with a pore size of 0.45 μm, and a membrane transfer time of 1 h. After completion of the transfer, the membrane was stained with Ponceau staining reagent, and the result of the membrane transfer was observed. Blocking was performed with the following steps: the membrane was completely immersed in 3% BSA-TBST with shaking at room temperature for 30 min. Primary antibody incubation was as follows: the primary antibodies included lysosome-associated membrane protein-2 (LAMP2) anti-goat polyclonal antibody (1:200, sc-8100, Santa Cruz Biotech, USA); cathepsin L anti-goat polyclonal antibody (CTSL1, 1:200, sc-6498, Santa Cruz Biotech, USA); and β-actin (1:5000, YM3028, Immunoway, USA). After incubating at room temperature for 10 min, the membrane was incubated at 4°C overnight. Washing was performed as follows: the membrane was washed with TBST five times (3 min for each wash). Secondary antibody incubation was performed as follows: the secondary antibody was diluted with 5% nonfat dry milk-TBST, and the goat anti-rabbit IgG (H+L) HRP (1:20000, Cat No. S001, tdybio, Beijing, China) was added to the NC membrane. After shaking at room temperature for 40 min, the membrane was washed: the membrane was washed with TBST six times (3 min for each wash). ECL reagent (Cat No. WBKLS0500 Millipore, USA) was added to the membrane and allowed to react for 3-5 min, followed by film exposure for 2 min, film developing for 2 min, and fixation.

### Intracellular lysosomal pH detection, cathepsins L activity assay and analysis of autophagic flux

4.8.1 Standard curve preparation: Normal AR42J cells were seeded in 96-well plates for 24h, and then incubated in pH gradient EMS buffer (20 mM NaCl, 110 mM KCl, 20 mM EMS, HCl/NaOH to adjust the pH) containing 10 μM monensin (Selleck, China), 20 μM nigericin (MedChemExpress, USA) and LysoSensor Yellow/Blue DND-160 fluorescent dye (Thermo, Shanghai, China). Five pH gradients were set at pH 4.0, pH 4.5, pH 5.0, pH 5.5, pH 6.0. After incubation for 30 minutes at room temperature, the standard curve of pH-fluorescence intensity ratio was plotted by measuring the fluorescence intensity of 340 nm to 380 nm (F340 / 380) with a multifunctional microplate reader (TECAN, Switzerland).

4.8.2 The experimental group cells were inoculated into 96-well plates and the culture medium was replaced with culture medium containing LysoSensor Yellow / Blue DND-160. After incubation for 30 minutes at room temperature, the fluorescence intensity ratio (F340 / 380) was measured at 340 nm and 380 nm, and then the cell lysosomal pH were calculated corresponding to the standard curve.

4.8.3. Cells were lysed and treated with reaction buffer that was included in the kits used for cathepsin L (cat. no. ab65306; Abcam), and 10 mM fluorogenic Ac-FR-amino-4-trifluoromethyl coumarin (AFC) substrate, which is the preferred cathepsin L substrate according to the manufacturer’s protocol. Fluorescence was measured using multifunctional microplate reader (TECAN, Switzerland), at an excitation wavelength of 400 nm and an emission wavelength of 505 nm.

4.8.4. To analyse autophagic flux, AR42J cells were transfected with a tandem fluorescent mRFP-GFP-tagged LC3 plasmid using Lipofectamine 3000. The expression of GFP and mRFP was visualized with laser confocal microscope (A1R, Nikon, Japan). Images were acquired using FV10-ASW3.0 software. Yellow (merge of GFP signal and RFP signal) puncta represented early autophagosomes, while red (RFP signal alone) puncta indicate late autolysosomes. Autophagic flux was evaluated by the colour change of GFP/mRFP.

### Statistical methods

The data are represented as the mean ± standard deviation, and differences of *P* < 0.05 were considered statistically significant. The miRNA and mRNA with differential expression were compared using the frequency-directed run-length (FDR) test, and FDR < 0.05 was considered to indicate differential expression. All abovementioned statistical analyses were carried out using SPSS 13.0 software.

## Abbreviations

AP: Acute pancreatitis
CTSB: Cathepsin B
CTSL and CTSL1: Cathepsin L
LAMP2: Lysosome-associated membrane protein 2
TLC-S: Taurolithocholic acid 3-sulfate
SP-PIR-KEYWORDS: Swiss-Prot and ProteinInformation Resource KEYWORDS
KEGG: Kyoto Encyclopedia of Genes and Genomes
GO-MF: gene ontology molecular function
GO-CC: gene ontology cellular components
GO-BP: gene ontology biological process
PCR: polymerase chain reaction
HMGB1: high mobility group box 1
HEXB: hexosaminidase B
RGD: Rat Genome Database
qPCR: quantitative PCR
PBS: phosphate-buffered saline
AFC: Ac-FR-amino-4-trifluoromethyl coumarin
FDR: frequency-directed run-length
